# Atypical clinical presentation of a severe Tumor Necrosis Factor Receptor-associated Periodic Syndrome (TRAPS) without mutation in the TNFRSF1A gene and good response to anakinra. Case report of a ten year old girl with fever, skin edema and abdominal pain (AID-registry)

**DOI:** 10.1186/1546-0096-13-S1-P43

**Published:** 2015-09-28

**Authors:** F Hamsen, C Müntjes, W Kampmann, B Schweiger, U Neudorf, E Lainka

**Affiliations:** 1 University Children's Hospital, Pediatric Rheumatology, Essen, Germany; 2 Christliches Kinderhospital Osnabrück, Clinic of Pediatrics, Osnabrück, Germany; 3 University Hospital Essen, Institute of Diagnostic and Interventional Radiology and Neuroradiology, Essen, Germany

## Background

Tumor Necrosis Factor Receptor Associated Periodic Syndrome (TRAPS) is a hereditary autoinflammatory syndrome characterized by reccurrent episodes of fever and localized inflammation. It is characterized by recurrent fever accompanied by abdominal pain, pleuritis, migratory skin rashes, fasciitis, headache, conjunctivitis and periorbital edema.

## Case report

We report about a 10 year old girl from Romania who suffered from fever, skin edema and abdominal pain. She was no longer able to walk because of pain and took NSAIDs and tramadol. The right arm was swollen. In the short past she suffered from pneumonia and took cefuroxime oral. Five years ago tuberculosis was diagnosed and completely healed up by a triple therapy for 6 months. A few months ago periorbital edema were described in Romania. She spent 2 months in hospital. Infectiological, oncological and other causes for fever were excluded and no diagnosis was found but prednisolone improved edema and fever. Blood test showed leukocytosis, thrombocytosis and high elevated CrP and SAA. S 100 A12 Protein was normal. Genetic analysis for hereditary recurrent fever syndromes (HRFs) showed no mutations in the four commonest genes. There is a negative family history for periodic fever syndromes or rheumatic diseases. After reduction of prednisolone symptoms immediately recurred. Etanercept was unsuccessful. After literature research [1] we diagnosed TRAPS without mutation and started a therapy with anakinra (IL-1 inhibitor). The response was prompt and dramatic. Now the patient gets anakinra daily s.c. and she had no longer fever or edema. Because of problems with the health insurance and because of the off-label indication for TRAPS she had to interrupt the medication for a few days and the symptoms came back again. Now she takes anakinra since 3 months.

## Conclusions

TRAPS should be considered in cases like this without mutation. Anakinra is a good therapeutic option; actually the use is off-label.

## Acknowledgements

The AID-Registry is funded by the BMBF since 2009 (01GM08104, 01GM1112D).

## Consent to publish

Written informated consent for publication of their clinical details was obtained from the patient/parent/guardian/relative of the patient.
